# Brain–Computer Interfaces: Toward a Daily Life Employment

**DOI:** 10.3390/brainsci10030157

**Published:** 2020-03-09

**Authors:** Pietro Aricò, Nicolina Sciaraffa, Fabio Babiloni

**Affiliations:** 1 Department of Molecular Medicine, Sapienza University of Rome, Piazzale Aldo Moro, 5, 00185 Rome, Italy; nicolina.sciaraffa@uniroma1.it (N.S.); fabio.babiloni@uniroma1.it (F.B.); 2 BrainSigns srl, Lungotevere Michelangelo 9, 00192, Rome, Italy; 3 IRCCS Fondazione Santa Lucia, Neuroelectrical Imaging and BCI Lab, Via Ardeatina, 306, 00179 Rome, Italy; 4 College of Computer Science and Technology, Hangzhou Dianzi University, Hangzhou 310018, China

**Keywords:** passive brain–computer interface (pBCI), EEG headsets, daily life applications

## Abstract

Recent publications in the Electroencephalogram (EEG)-based brain–computer interface field suggest that this technology could be ready to go outside the research labs and enter the market as a new consumer product. This assumption is supported by the recent advantages obtained in terms of front-end graphical user interfaces, back-end classification algorithms, and technology improvement in terms of wearable devices and dry EEG sensors. This editorial paper aims at mentioning these aspects, starting from the review paper “Brain–Computer Interface Spellers: A Review” (Rezeika et al., 2018), published within the Brain Sciences journal, and citing other relevant review papers that discussed these points.

A brain–computer interface (BCI) was originally defined as “a communication system in which messages or commands that an individual sends to the external environment do not pass through the brain’s normal output pathways of peripheral nerves and muscles”. For example, in an electroencephalogram (EEG)-based BCI, the messages can be directly decoded by specific EEG features [1]. In 2012, Wolpaw and Wolpaw [2] widened the meaning of the brain-computer interface, defining it as “a system that measures Central Nervous System (CNS) activity and converts it into artificial output that replaces, restores, enhances, supplements, or improves natural CNS output and thereby changes the ongoing interactions between the CNS and its external or internal environment”, suggesting the possibility of employing this technology for different applications and targeting different kind of potential users, starting from completely locked-in people (e.g., amyotrophic lateral sclerosis, ALS), in which BCI can be used in its original meaning, or in other words in an “active” way (Active BCI), in which the user voluntary modulates his/her brain activity to generate a specific command on the surrounding environment (i.e., to replace and/or restore lost or impaired muscular abilities, [3,4,5]), coming to healthy users in daily life applications. In particular, BCI for healthy users could be used to enhance human–surroundings interaction. In this regard, the BCI (i.e., passive BCI, pBCI, [6,7,8,9,10,11,12,13]) is able to derive its outputs from arbitrary brain activity arising without the purpose of voluntary control (i.e., implicit information on the user states), for example, workload, attention, emotion, and most in general task-induced states that can only be detected with weak reliability using conventional methods such as subjective (e.g., questionnaires) and/or behavioral (e.g., reaction times) measures [14]. Systems based on pBCIs can directly use in a closed loop this information about the user states to automatically modify the behavior of the interface that the user is interacting with (i.e., adaptive automation), or just to inform, even in real-time, the user himself/herself or other people about dangerous human behaviors (e.g., overload [15], or loss of vigilance [16,17]) that could increase the human error probability and consequently induce possible unsafe situations. 

Several giant leaps have been made in the BCI field in the last years, from several points of view. For example, many works have been produced in terms of *front-end* graphical user interfaces (GUIs), as deeply reported in the review paper “Brain–Computer Interface Spellers: A Review” recently published in the Brain Sciences journal. In this regard, “*throughout the years, scientists have worked on spelling systems to make them faster, more accurate, more user-friendly, and, most of all, able to compete with traditional communication methods”* [18]. 

In this particular regard, a huge effort has been made even in *back-end* algorithms (i.e., classification techniques) running under BCI systems [19], allowing for high discrimination accuracy (e.g., target vs. no-target, low workload vs. high workload) together with high information transfer rates (ITRs) and by using less and less features (i.e., EEG sensors). In this regard, machine-learning and deep learning approaches based on the analysis of physiological data went through a rapid expansion in the last decade since such methodologies are able to provide the means to decode and characterize task relevant brain states (i.e., reducing from a multidimensionality to one dimensionality problem) and to distinguish them from non-informative brain signals (i.e., to enhance Signal to Noise Ratio). In this regard, Aricò and colleagues have published a few review papers demonstrating the maturity and effectiveness of this kind of technique by testing BCI systems in daily life applications [20,21]. Figure 1 shows BCI concept and related potential fields of application. 

Last, but not least important, enhancement in technology is related to EEG recording headsets that could finally allow BCI systems to enter the market, especially for daily life applications. In recent years, many companies have been moving to develop more wearable and minimally invasive biosignal acquisition devices. With particular regard to EEG systems, current effort is being made to develop dry sensors (i.e., not requiring any conductive gel), or to eventually use water-based technology instead of the classic gel-based technology, allowing high signal quality and higher comfort (e.g., [22]). There is a common opinion that gel-based electrodes still have to be considered the gold standard [23,24], however, the gap between wet and dry electrodes is being more and more reduced [25]. Several attempts are already present in the literature about the comparison and validation of these innovative dry EEG electrodes. In this regard, recently Di Flumeri and colleagues [25] published a paper aiming to assess the level of maturity achieved by the EEG dry electrodes industry by comparing three different types of dry electrodes with traditional ones (i.e., gel-based). The results of this work highlighted the high level of quality achieved by dry EEG solutions, since all the tested electrodes were able to guarantee the same quality levels of the wet electrodes, allowing at the same time significantly reduced times of montage and improvement in the users’ comfort.

In conclusion, because of the leaps and bounds performed in terms of front-end interfaces and back-end algorithms of BCIs, and the huge technology improvement in terms of wearable devices and dry EEG sensors, we can infer that BCIs are not too far from leaving the labs, and entering the market as a new consumer product.

## Figures and Tables

**Figure 1:**
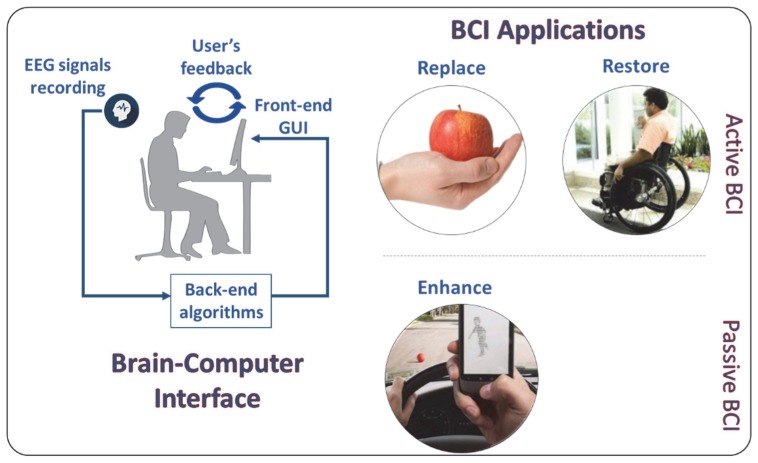
The brain–computer interface concept and related applications that could be realized in an *Active*, and a *Passive* meaning.
